# Strategies for high-throughput focused-beam ptychography

**DOI:** 10.1107/S1600577517009869

**Published:** 2017-08-08

**Authors:** Chris Jacobsen, Junjing Deng, Youssef Nashed

**Affiliations:** a Advanced Photon Source, Argonne National Laboratory, USA; b Department of Physics and Astronomy, Northwestern University, USA; c Chemistry of Life Processes Institute, Northwestern University, USA; d Mathematics and Computer Science Division, Argonne National Laboratory, USA

**Keywords:** ptychography, ptychographic resolution gain, high throughput

## Abstract

X-ray ptychography is being utilized for a wide range of imaging experiments with a resolution beyond the limit of the X-ray optics used. Introducing a parameter for the ptychographic resolution gain *G*_p_ (the ratio of the beam size over the achieved pixel size in the reconstructed image), strategies for data sampling and for increasing imaging throughput when the specimen is at the focus of an X-ray beam are considered. The tradeoffs between large and small illumination spots are examined.

## Abstract 2

X-ray ptychography is being utilized for a wide range of imaging experiments with a resolution beyond the limit of the X-ray optics used. Here, strategies for data sampling and for increasing imaging throughput when the specimen is at the focus of an X-ray beam are discussed, and the tradeoffs between large and small illumination spots are examined.

## 1. Introduction   

Ptychography (Hoppe, 1969 ▸) involves the use of overlapping coherent illumination regions on a specimen and the collection of diffraction data from each illumination spot, followed by reconstruction of an image. Following initial experimental demonstrations of ptychography (Rodenburg *et al.*, 2007 ▸; Thibault *et al.*, 2008 ▸) and related methods (Chapman, 1996 ▸), X-ray ptychography is finding increased utilization in X-ray microscopy because it can be used to deliver amplitude and phase-contrast images beyond the resolution limit of the coherent beam size. It does so without the small isolated specimen limitations of X-ray coherent diffraction imaging (Miao *et al.*, 1999 ▸) which are intrinsic to the use of finite support iterative phase retrieval (Fienup, 1978 ▸) unless one uses a spatially restricted coherent beam to satisfy the support constraint (Abbey *et al.*, 2008 ▸) (which begins to look like ptychography if one scans the beam).

## 2. Discussion   

**Figure 1:**
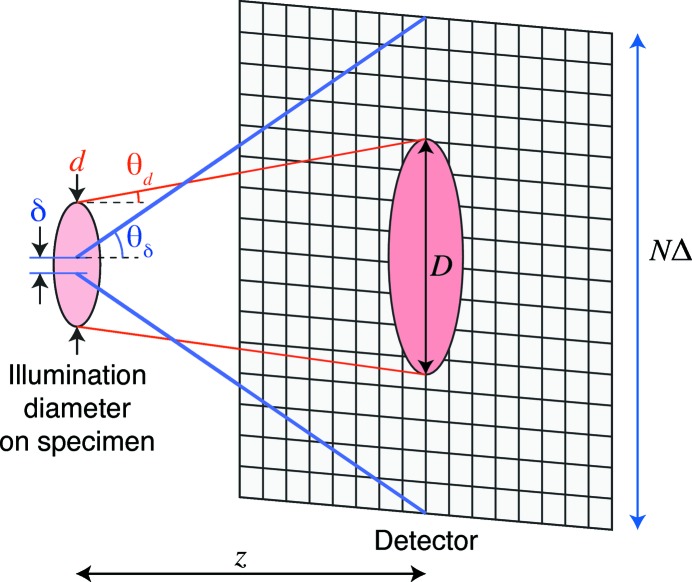
Data sampling in ptychography. The specimen is illuminated by beam spots of diameter *d* with divergence 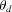, leading to an incident illumination diameter *D* on the detector. The desired half-period pixel width δ (the limit of resolution) implies a diffraction numerical aperture 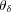 out to the edges of the detector.

Because ptychography requires mostly coherent beams, and involves point-by-point scanning, it is usually regarded as a low-throughput imaging method even though several higher throughput examples exist (Guizar-Sicairos *et al.*, 2014 ▸; Holler *et al.*, 2014 ▸). We consider here the factors that can be used to increase that throughput, characterizing them in terms of the ptychographic resolution gain 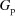 which we define as the ratio between the desired reconstructed image pixel size^1^ δ and the diameter *d* of the coherent beam spot that is scanned across the specimen, or
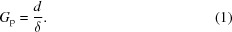
In order to have the possibility of a square half-period pixel size of δ using an illumination wavelength λ, Fig. 1 ▸ shows that one must record first-order diffraction out to an angle (in the small-angle approximation of 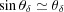) of
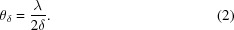
This requires coherent superposition between waves with a path length difference from the top and bottom edges of the illumination spot to a distant detector of 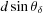, or a number *m* of wavelengths of longitudinal coherence (Spence *et al.*, 2004 ▸; van der Veen & Pfeiffer, 2004 ▸; Enders *et al.*, 2014 ▸) of
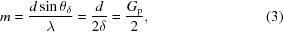
where we have again used the small-angle approximation. Therefore the spectral bandwidth should be
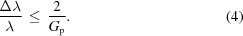
Illumination from the coherent illumination spot of diameter *d* will diffract out by a semi-angle^2^ of 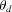 = 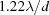 to cover a diameter *D* on the detector of
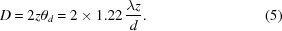
The signal from small features within the illumination spot is recorded on a detector with *N* pixels (each of width Δ) on a side, so that it subtends a semi-angle of
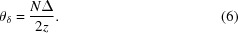
The illumination spot will therefore be spread out over a number of detector pixels 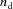 of
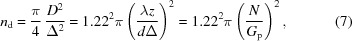
where the final expression uses equations (1), (2) and (5). Finally, because the last two pixels on the detector must be able to record fringes caused by interference from points at the top and bottom of the illumination spot *d* with Nyquist sampling, we must have 2*m* pixels from the detector center to the edge or 4*m* pixels overall in each dimension. In other words, we find that the minimum number of detector pixels 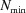 is given by
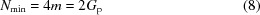
where we have used equation (3). We note that ptychography can be performed with reduced detector sampling, though at a cost of image fidelity (Edo *et al.*, 2013 ▸) or of finer real-space sampling (Batey *et al.*, 2014 ▸; da Silva & Menzel, 2015 ▸) which effectively corresponds to larger values of the overlap factor *o* discussed below.

Achieving high resolution in ptychography requires the detection of scattering out to large angles, which is determined in part by the specimen’s optical properties at the chosen X-ray wavelength and the number of photons 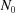 used to illuminate each pixel (Glaeser, 1971 ▸; Sayre *et al.*, 1977 ▸; Schropp & Schroer, 2010 ▸). While high resolution can also be aided by having high-spatial-frequency content in the illuminating beam if a larger focal spot (larger value of 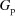) is used (Guizar-Sicairos *et al.*, 2012 ▸), we assume that the dominating factor is the required photon density per area, 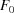 = 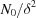, independent of the ptychographic spatial resolution gain 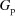. If *a* is the center-to-center position increment between illumination spots of diameter *d*, it is recommended to use an overlap factor 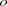 = 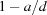 with 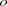 ≃ 0.6 for robust reconstructions (Bunk *et al.*, 2008 ▸) [this parameter has also been used in connection with continuous scan ptychography (Deng *et al.*, 2015*a* ▸)]. Imaging of a square area *A* then requires the use of a number 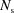 of illumination spots given by
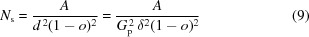
if one ignores incomplete illumination at the edges. Imaging the area *A* then requires a net photon count 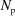 of
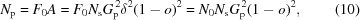
where we have used equation (9) to arrive at the final result. If the source delivers a coherent flux of 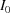 photons per second, the exposure time 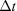 for each of the 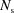 illumination spots (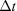 equals the transit time per distance *a* in the case of continuous scanning) can be found from 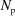 = 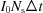, leading to an exposure time per illumination spot of
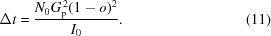
Because the coherent flux 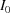 is an intrinsic property of the source [with the caveat that one can obtain higher coherent flux from a broad-band source as indicated by equation (4)], it is unchanged by the size *d* of the spot into which the coherent flux is delivered; that is, 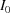 does not depend on the ptychographic spatial resolution gain 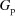.

From the above, we see that decreasing the ptychographic spatial resolution gain 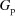 (that is, using smaller coherent illumination spots *d*) has several implications:

(i) Decreasing 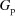 means detectors with fewer pixels 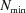 can be used, as can be seen directly from equation (8).

(ii) Decreasing 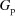 leads to relaxed requirements for the monochromaticity 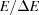 in the illumination, as can be seen from equation (4). When using large-bandwidth X-ray sources, this could allow one to use multilayer monochromators with bandpass 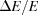 ≃ 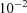 as compared with crystal monochromators with bandpass 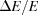 ≃ 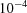, thus leading to a usable flux gain increase of 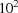. This has already been demonstrated as a route to increased throughput in ptychography (Enders *et al.*, 2014 ▸).

(iii) Decreasing the gain 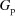 has the effect of increasing the fractional number of detector pixels within which the incident beam is recorded, as can be seen from equation (7) divided by 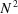. Since the incident beam is often much stronger than the fraction of the beam scattered by the sample, putting the incident beam into a larger fraction of detector pixels reduces demands on the dynamic range of the detector. This is in contrast to the case of far-field coherent diffraction imaging of weakly scattering objects like biological cells, where the dynamic range required of the detector can be in excess of 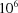:1 so that multiple detector exposures must be acquired with various exposure times and direct-beam absorber positions (Shapiro *et al.*, 2005 ▸).

(iv) When smaller illumination spots *d* are used with smaller ptychographic gain 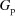, one has the option of simultaneously acquiring high-spatial-resolution scanning microscope images using other contrast modes, such as fluorescence from elemental content (Schropp *et al.*, 2012 ▸; Deng *et al.*, 2015*b* ▸, 2017 ▸).

(v) Decreasing 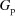 is associated with shorter exposure times per diffraction pattern recording, as can be see from equation (11). This means that higher detector frame rates are required if one uses smaller values of 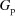. However, the net ‘information rate’ to be transferred by the detector of total pixels per second, or 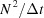, is not affected as can be seen by the fact that both 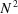 [from the square of equation (8)] and the exposure time 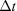 [from equation (11)] depend on 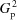, thus canceling out any dependence on 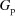. In other words, the total amount of data to be saved or transferred is the same in optimized experiments, independent of the ptychographic gain 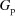. We have not accounted for factors such as thermal noise or amplifier readout noise which might be present in charge-integrating detectors *versus* photon-counting detectors.

(vi) Finally, if there is an overhead time associated with the collection of signal from each of the 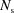 illumination spots, reducing 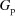 and thus increasing 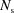 [equation (9)] will lead to longer scan times. However, continuous-scan methods largely remove this overhead time between pixels (Pelz *et al.*, 2014 ▸; Deng *et al.*, 2015*a* ▸); in raster scans, the overhead between scan lines depends both on the step distance (favoring small 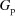) and data transfer overheads (favoring large 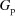).

These advantages of using small illumination spots or small values of ptychographic spatial resolution gain 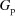 can be compelling for certain applications. Finally, we note that increased experimental throughput should be coupled with fast computing; one example is to use efficient parallelization schemes for ptychographic image reconstruction (Nashed *et al.*, 2014 ▸).

## Footnotes

1 Of course, the achieved image resolution might be poorer than the reconstructed image pixel size δ due to factors such as illumination strength and intrinsic specimen scattering as will be noted below.

2 If one instead assumes a circular focusing optic with a numerical aperture of , then *d* refers to the diameter of the Airy disk of the focus; the numerical factor of 1.22 which is slightly modified depending on central stops, or non-circular optics, plays only a minor role in the discussion that follows.
